# The emergence of ethical issues in the provision of online sexual health outreach for gay, bisexual, two-spirit and other men who have sex with men: perspectives of online outreach workers

**DOI:** 10.1186/s12910-017-0216-7

**Published:** 2017-11-03

**Authors:** Sophia Fantus, Rusty Souleymanov, Nathan J. Lachowsky, David J. Brennan

**Affiliations:** 1 Factor-Inwentash Faculty of Social Work, University of Toronto, 246 Bloor Street West, Toronto, ON M5S 1V4 Canada; 2 School of Public Health and Social Policy, University of Victoria, Victoria, BC Canada; 3 Ontario HIV Treatment Network Research Chair, Factor-Inwentash Faculty of Social Work, University of Toronto, 246 Bloor Street West, Toronto, ON M5S 1V4 Canada

**Keywords:** Online sexual health outreach, Ethics, Outreach workers, GB2M, HIV prevention, Boundaries, Confidentiality

## Abstract

**Background:**

Mobile applications and socio-sexual networking websites are used by outreach workers to respond synchronously to questions and provide information, resources, and referrals on sexual health and STI/HIV prevention, testing, and care to gay, bisexual and other men who have sex with men (GB2M). This exploratory study examined ethical issues identified by online outreach workers who conduct online sexual health outreach for GB2M.

**Methods:**

Semi-structured individual interviews were conducted between November 2013 and April 2014 with online providers and managers (*n* = 22) to explore the benefits, challenges, and ethical implications of delivering online outreach services in Ontario, Canada. Interviews were digitally recorded and transcribed verbatim. Thematic analyses were conducted, and member-checking, analyses by multiple coders, and peer debriefing supported validity and reliability.

**Results:**

Four themes emerged on the ethical queries of providing online sexual health outreach for GB2M: (a) managing personal and professional boundaries with clients; (b) disclosing personal or identifiable information to clients; (c) maintaining client confidentiality and anonymity; and (d) security and data storage measures of online information. Participants illustrated familiarity with potential ethical challenges, and discussed ways in which they seek to mitigate and prevent ethical conflict.

**Conclusions:**

Implications of this analysis for outreach workers, researchers, bioethicists, and policy-makers are to: (1) understand ethical complexities associated with online HIV prevention and outreach for GB2M; (2) foster dialogue to recognize and address potential ethical conflict; and (3) identify competencies and skills to mitigate risk and promote responsive and accessible online HIV outreach.

## Background

The increasing accessibility of the Internet and the ubiquity of handheld devices, such as smartphones and tablets, have significantly changed health service delivery [1, 2]. Formal online therapy programs such as e-therapy or e-counseling use communication tools that are either synchronous (e.g., chat) or asynchronous (e.g., short message services, texting) [1, 3–6]. These tools increase accessibility to underserviced or geographically rural areas [7, 8], and to those struggling with physical disabilities, social anxieties and phobias, and body image issues [9–11]. Research shows that client outcomes are often as successful as face-to-face services [12]; clients report being highly satisfied with online therapy, forming intimate, engaging, and strong relationships with service providers [13–16].

The rapid expansion of computers and mobile devices has also reshaped how patients are acquiring health information and resources [17–19]. In 2012, an estimated 72% of adults living in the United States who use the Internet reported looking up online health information within the past year [20]; in 2015, 62% of smartphone owners used their phones to look up information about a health condition [21]. Internet-based health resources have particularly facilitated access to sexual health information and counseling, offering low cost, efficient practice, and opportunities for interactive, non-discriminate and anonymous care [22]. In the 2010–2011 Teen Health and Technology study administered to 13–18 year olds in the U.S. (*n* = 5542), findings demonstrated that sexual minority youth have significantly higher rates (78%) of seeking online sexual health information than their heterosexual peers (19%). Privacy and curiosity were the main determinants of seeking online resources [23], potentially attributable to stigma and shame across sexual minority populations.

Online health resources have rapidly expanded to incorporate HIV/STI prevention strategies and sexual health education specifically for gay, bisexual, two-spirit (for a definition, please see www.lgbtqhealth.ca/community/two-spirit.php) and other men who have sex with men (herein referred to as “GB2M,” inclusive of cisgender, two-spirit and transgender men). In the 2012 Sex Now Survey, conducted among gay and bisexual men in Canada (*n* = 8388), 71.53% of participants reported an intention to use Internet-based STI/HIV testing [24]. Benefits associated with these programs, according to participants, included privacy, convenience and flexibility [24]. The authors suggest that Internet-based testing has the potential to reach diverse subpopulations of GB2M, including men at risk of STIs and HIV and those facing current barriers to testing, such as access, geographical distance, wait times and/or fears of privacy and anonymity [24].

GB2M have been early adopters of online technologies to develop social and sexual connections [25–27]. Mobile applications and socio-sexual networking websites are frequently used to seek sexual, social and romantic partners [28–31]. More recently, these mobile applications and socio-sexual networking websites have been employed to promote sexual health information and enhance HIV outreach and prevention among GB2M [31–39]. Unlike online sexual health information or Internet-based testing programs, online sexual health outreach involves trained staff or volunteers who use online sites and apps to respond synchronously to questions or provide information, resources and referrals regarding sexual health and STI/HIV prevention, testing and care. Online sexual health outreach is an individualized one-on-one conversation conducted by a trained front-line worker. Online outreach workers are community members who are hired or volunteer primarily with AIDS service organizations, public health units and community-based partnerships, and are trained to answer questions, provide information, and offer referrals to local agencies and services.

Online sexual health services are highly acceptable to GB2M [39–41]. Preliminary research exploring the effectiveness of online sexual health outreach has demonstrated an increase in HIV/AIDS knowledge, self-efficacy and condom use among men who have sex with men [42, 43]. Notwithstanding the demonstrated benefits of online sexual health outreach, there are a number of potential ethical considerations that are critical to address. The advent of online platforms, such as social media and socio-sexual networking websites, has introduced ethical dimensions that may influence the use and impact of online outreach. Recent studies examining both synchronous and asynchronous online sexual health outreach suggest that service users have expressed concerns with regard to confidentiality, privacy and issues related to access, usage and storage of their personal information [44, 45]. To our knowledge, there has been no empirical research from the perspective of service providers to understand how they perceive the emergence of ethical issues in online sexual health outreach or how they have considered and/or employed strategies to mitigate potential risks.

Online outreach is a relatively new platform that is run and managed by community members and volunteers (who may or may not hold affiliations with allied health professional regulatory bodies). There are limited policies, standards and procedures in place to help guide the provision of online therapeutic support, case management and sexual health information. Consequently, research is needed to understand the experiences of online outreach workers and their perspectives of ethical practice issues that may emerge in their work. Elucidating online outreach experiences may help identify and develop future guidelines to help to enhance the provision of online sexual health outreach for GB2M and other populations. Thus, the purpose of this study was to investigate the perspectives of online outreach workers to understand how they describe and identify ethical issues that emerge in the delivery of online sexual health outreach for GB2M.

Objectives of this study were to: (1) examine how online outreach workers describe and identify ethical issues that have emerged in their delivery of online outreach services for GB2M in Ontario; (2) understand the ways in which online outreach workers consider issues of safety and confidentiality in the delivery of their practice; and (3) address strategies employed by online outreach workers and managers in which to consider and mitigate emerging ethical dilemmas that they have described encountering in their practice. In turn, as online sexual health outreach among GB2M is being demonstrated as an important and potentially effective means of providing HIV/STI counseling and intervention, the ethical implications are critical to understand and address.

## Methods

This paper presents qualitative findings from a larger mixed-methods study designed to examine online sexual health outreach among GB2M. The quantitative survey was focused on GB2Ms’ experience with online sexual health outreach [43] and the qualitative findings reported here were gathered from interviews with agencies that provide online outreach to GB2M. This study included an active Community Advisory Board (CAB) with a diverse representation of GB2M (race, HIV-status, geographical location).

### Recruitment

From November 2013 to April 2014, outreach providers and managers at AIDS service organizations (ASOs), community-based organizations (CBOs), and public health units were recruited to complete a 1-h in-person/telephone semi-structured interview to explore in-depth their experiences with, and perspectives on, delivering online sexual health outreach for GB2M in Ontario, Canada. Participants came from diverse geographical locations, which are summarized for reporting as either the Greater Toronto Area (GTA), Ontario (Outside the GTA) or outside of Ontario (including from the United States). This broad geographic sample was purposefully sought to engage: (1) with experts in the field; (2) with individuals who had specific experiences providing online sexual health outreach; and (3) with agencies who were known to have successful online outreach programs. Recruiting in this manner helped to inform our study objectives by broadening perspectives and engaging with those who have worked in established online sexual health outreach programs. Participants worked (paid or volunteer) and were involved or interested in online HIV prevention and outreach programs and services for GB2M. Participants were required to meet one of the following criteria: (1) currently providing online outreach services to GB2M; (2) interested in or planning to provide (but are not currently providing) online outreach services to GB2M; or (3) formerly provided or not planning to provide online outreach services to GB2M. Informants were recruited through our CAB as well as through e-mail listservs of the ASOs and CBOs funded by the AIDS Bureau at Ontario’s Ministry of Health or who were members of the Ontario Gay Men’s Sexual Health Alliance. Using targeted sampling, our recruitment strategy sought participants from diverse geographic locations, job positions and the type of provider (public health units versus community agencies). Participants were offered entry into a random draw to receive one of four prizes of $100.00 (CDN) as compensation for their time. This study was approved by the Research Ethics Boards of the University of Toronto and the University of Guelph.

### Data collection

All participants provided informed consent prior to the interviews, which were conducted in-person or over the phone by a single research assistant. The research assistant only contacted participants who expressed interest in the project. Before proceeding with the interviews, participants were informed about the purpose of the study, all possible risks and benefits and confidentiality and data storage measures. Participants verbally reviewed the informed consent with the research assistant and subsequently provided written consent.

Each interview consisted of a semi-structured interview guide with scripted probes to explore in-depth the providers’ experiences and perspectives of the (ethical) implications of online sexual health outreach programs and services for GB2M. Participants were asked about the issues, core values and principles associated with providing online sexual health outreach to diverse GB2M. Questions sought to identify the organizational structures, policies, or programs that support online sexual health outreach, and to assess challenges when providing online outreach. All interviews were recorded electronically and transcribed verbatim using a professional transcription service, which operated in accordance with a signed confidentiality agreement, and was certified by provincial and federal regulations regarding privacy. Prior to data analysis, all identifying information was redacted from the transcripts.

### Data analyses

This particular analysis focuses on the ethical implications of delivering online sexual health outreach services for GB2M in Ontario. Semi-structured interview questions, included: (a) can you please talk about issues of safety and confidentiality; (b) what other ethical concerns have emerged doing online outreach work; (c) what vulnerabilities do your online outreach services have; and (d) what kind of programming do you struggle to maintain? Added probes to help participants respond comprised: Do you use real names? Do you use logos or personal pictures? How do you protect your identity? Do you keep records of conversations, chats, referrals? What happens when sensitive and/or identifying data is disclosed? How do you think through using these sites as service providers and service users?

Questions were phrased in an open-ended manner to glean participants’ experiences and perspectives regarding issues that they identified as ethically ambiguous in their practice. However, some interview questions were more specific and attuned to particular ethical issues. These questions were framed with consideration of the research team’s prior conversations with community members and their observations in fields of practice. The research team and the project’s community advisory board assumed an active role in the design and implementation of this research study. Consequently, issues specifically regarding safety and confidentiality, as well as questions involving the use of real names, logos and stored conversation records, originated from dialogues with the community advisory board concerning relevant issues in the practice field. The content of these research questions was also determined in conjunction with previous scholarship exploring of the use of online psychotherapy. Empirically-based findings have consistently shown that ethical issues surrounding confidentiality, dual relationships, unanticipated contact and record keeping are identifiable concerns among both service users and service providers when utilizing online mechanisms for therapeutic support [46]. Although the participant population in the current study is not comprised of trained and/or regulated healthcare professionals, it was important that the interview questions were guided and directed, in part, by evidence-informed research across similar fields of practice.

Thematic analyses were conducted using NVivo10 data analysis software [47]. Inferences from the data were drawn following inductive analysis and closely grounded in the objectives of this paper. We compared all of the data and grouped similar narratives into themes. The community-based focus of our research entailed that data analysis incorporated extensive community involvement. The preliminary findings were reported to the CAB, and member-checking with the study team, persons from ASOs, CBOs and CAB members, supported validity and data trustworthiness. Only anonymized excerpts of the transcripts and aggregated findings were shared with the research team and CAB. During these meetings, disagreements in coding were resolved by reaching consensus among the research team and members of the CAB.

## Results

### Sample characteristics

Twenty-two respondents participated in this study. Eighteen participants worked in AIDS service (ASOs) or community-based organizations (CBOs), and four participants worked in public health units. Eighteen participants identified as front-line workers, two were managers and two were volunteers. Five interviews were conducted with participants outside of Ontario (four of which were in the United States). Of those within Ontario, seven were from the Greater Toronto Area (labeled: GTA) and 11 were located outside the GTA (labeled: Ontario). Nineteen participants were currently providing online outreach and three had previous experience doing so.

Overall, four themes emerged among online outreach workers and managers when conducting online sexual health outreach: (1) managing personal and professional boundaries with clients; (2) disclosing personal or identifiable information to clients; (3) maintaining client confidentiality and anonymity; and (4) security and data storage measures of online information. Each theme delineates: (1) an emerging ethical dilemma and (2) the strategies employed to mitigate risk. Participants generally indicated an understanding and familiarity of potential ethical challenges, and discussed ways in which they sought to mitigate and prevent ethical conflict. Although these four themes do consist of overlapping ethical issues, they were identified and described by participants as separate issues, consisting of unique strategies to mitigate potential risks. Each theme will be framed within the context of previous empirical scholarship in clinical ethics to help discern the potential ethical issues emerging in online outreach for GB2M. Owing to limited empirical research on issues in online outreach, findings may help to subsequently identify and develop ethical frameworks and directives to help guide this emerging practice.

### Managing personal and professional boundaries with clients

Participants considered how the provision of online sexual health outreach has introduced complex boundaries, whereby providers face real or potential conflict between their professional duties and their prospective or pre-existing social, sexual and/or romantic online and/or offline relationships. The issue of deciphering professional and personal boundaries in practice stems from the potential emergence of dual relationships, an ethical dilemma that may engender risks of client exploitation, power differentials, and a lack of objectivity and professionalism. Scholarship that has explored the use of online technologies has shown that community-based outreach workers often assume dual roles in their work, claiming both personal and professional relationships with their clients. Their personal connection to their community is often perceived as a way in which to facilitate knowledge of and familiarity with community resources and supports for service users [48]. To meet the needs of the community, service providers are often insiders, both living and working within the communities they serve.

For online sexual health outreach workers in this study, interactions between service users and providers were perceived as being professionally and personally blurred [48]. Blurred boundaries may occur due to the intersections between personal and professional uses of online apps and websites; online outreach workers commonly identify as GB2M, frequently using similar websites and apps to both personally and professionally connect with other GB2M. This may promote ambiguities with respect to maintaining professional boundaries between outreach workers and service users:

It’s hard to define between personal life and work life when it comes to outreach because it’s going to be part of your natural conversation if you want to make friends; it’s hard to describe the boundaries. You want to have fun, but you’ve got to work too. So, it’s just like, ah. (Outreach worker; GTA)

The simultaneous use of online applications and websites for outreach and for personal social, romantic and/or sexual interactions introduces ethical challenges, as online outreach becomes “that sort of playground you work for and you play in. You will meet people that you will find attractive” (Outreach worker; GTA). According to one program manager who supervises online outreach workers, the boundary

Gets blurry, and with the blurry lines they’re [outreach workers] not quite sure what is appropriate or what isn’t until they’re in it; it’s really confusing and super blurry because at the end of the day I have to remember that these are guys who are in the community. (Outreach worker; Outside Ontario)

Indistinguishable boundaries may also be inevitable when working in smaller or more geographically isolated communities, where the likelihood of individuals having pre-existing relationships or prior familiarity with one another may be expected. A participant described how, “the smaller of a town that you get, the more people know who you are; just by virtue of my job, people know who I am” (Outreach worker; Ontario). Participants emphasized maintaining a sense of professionalism when addressing boundaries: “Lots of people know each other. And if you see somebody that you know, we would ask that you just don’t contact them” (Outreach worker; Outside Ontario). Another participant advised that talking to somebody you know, “could get messy, so, I would say to avoid somebody you know personally, say, somebody you really know closely….I think that’s the only way you can get around that” (Outreach worker; Outside Ontario). As such, local provision of such services introduces unavoidable queries regarding the limits to professional boundary management.

To mitigate risks of violating one’s professional boundaries, participants delineated specific times when mobile applications are used for work purposes. A participant explained that

Work is work and personal time is personal time. As an outreach worker, you’re not cruising. You’re not looking for someone to hook up with and you’re not looking for someone for a later date. You’re doing this because you’re here to do a very important job. (Outreach worker; Outside Ontario)

Participants described creating “a really definitive line between your personal life and your professional life” (Outreach worker; Ontario) and learning “how to engage. I don’t bring work home, I don’t bring clients home” (Outreach worker; Outside Ontario).

Many agencies had specific policies about not meeting outreach users in-person or recalling their login information to connect with them after their professional outreach hours. Using online outreach simultaneously for professional and personal means was against most agency policies: “if you’re caught doing that [personal outreach] you will be suspended from the program” (Outreach worker; GTA). A participant indicated the usefulness of having agency guidelines: “I think when you’re starting out [doing online outreach], you do need to sit with your agency’s supervisor and come to that compromise of when is play time, when is work time” (Outreach worker; GTA). Other participants spoke of a “cool down” or an offline period in between utilizing applications and websites for personal and professional reasons: “we try to institute…and say if you’re having a conversation earlier try not to be online within the next 2 h or so. Try to give yourself some space from being tempted to go online and to engage people” (Outreach worker; GTA).

To mitigate potential boundary conflicts, participants also expressed being transparent with service users about their role as a provider rather than as a potential social or sexual connection:

You start chatting with a guy and something happens - there’s always a possibility that there will be a connection. You have to make it very clear to the person you’re communicating with that you’re online in your role as either an outreach worker or an outreach volunteer. (Outreach worker; Ontario)

Another participant articulated that, “If I do plan on doing it [online sexual health outreach], then I put a really strict line, like, this is just for work, or this is just for personal life” (Outreach worker; GTA).

Participants, however, also saw the benefit of using online mediums for both personal and professional means, as it “actually informs my professional life around understanding how people react and discuss; it’s important that I learn that by doing it in my personal life as well” (Public health; GTA). Another participant suggested that, “it’s actually beneficial to our program if the volunteers are familiar with the sites so that they can engage with guys in a way that is comfortable for those other guys” (Outreach worker; GTA). Participants, overall, articulated diverse perspectives at both the individual and agency levels in understanding and addressing boundaries regarding the use of mobile applications and socio-sexual networking sites for both professional (online outreach) and personal means. Participants in this study articulated thoughtful responses when thinking about ways in which to manage and mitigate potential dual relationships that may easily evolve online.

### Disclosing personal or identifiable information to clients

The practice of online sexual health outreach by community members has led to ethical ambiguity regarding whether and how to disclose personally identifiable information to service users. Although there is no standard guideline that determines whether and how a service provider should disclose personal information to clients, the decision to self-disclose is often made in accordance with how such disclosure may benefit the therapeutic relationship and the client’s treatment plan [49]. Thus, ethical principles of beneficence and non-maleficence, defined as the act to promote benefits and to avoid harm, respectively [49], facilitate this decision-making process. Participants in the current study upheld diverse perspectives on what aspects to disclose, and the potential tensions that arise between building rapport and being too personal or identifiable. While it may be difficult to foreshadow the benefits or harms that may emerge as a result of self-disclosure, the manner in which service providers consider aspects of disclosure may importantly help navigate the manifestation of these ethical issues [49].

However, in online outreach, disclosure may transpire quite differently than in face-to-face service provision. Participants described what seemed to be different levels of disclosure, issues that ranged from disclosing one’s name or age to issues surrounding HIV status and sexual practices. Some participants did not view disclosure of demographic information (e.g., name, picture) as a risk. Rather, the use of one’s real name was perceived to foster transparency and accountability: “I use my real name when I introduce myself; it’s really important for us to come forward and be as transparent as possible in that [online] environment.” (Outreach worker; Outside Ontario). Another participant explained the accountability of identifying oneself with their work: “I think on the one hand to me it cements the ownership of yes, whatever you’re saying to this person, even if it’s online or in this cyber digital world, it’s still traceable to you, so own it” (Outreach worker; Outside Ontario). Disclosing one’s name or organizational affiliation was also associated with a sense of legitimacy, as self-disclosure is, “to our advantage because that patient will know that we’re legitimate” (Public health; GTA).

Participants experienced a sense of comfort and public familiarity with their online outreach work: “I have no problems using my real name and my picture. I have an understanding of my community and respect for work and respect for my agency’s code of conduct.” (Outreach worker; GTA). Another participant reiterated this sentiment:

I’m very connected to the network around here and the community around here and I’m very involved in the non-profit organizations and social activism. So I’m comfortable with my information being out there. I’m comfortable with the fact that that might mean that somebody might locate me or find my information. (Outreach worker; Outside Ontario)

Participants implied that training and professionalism play a role in the decision of whether or not to disclose. Outreach workers who have been in the field for longer are typically more aware of how to protect their information, and have gained more comfort and familiarity with available community networks, and how to determine whether self-disclosure, in relation to name, age and geographical location, will be a benefit to the service user. A participant stated,

If a volunteer is doing the work, and he’s not comfortable giving out his personal information, we say to them too, you’re not set in stone in terms of giving your personal information; you don’t have to give your name, your age, where you come from, and all of that stuff. (Outreach worker; GTA)

Many agencies left the decision to disclose up to the individual outreach worker: “we have a recommendation around that, which is giving discretion to the volunteer of what to do in that situation” (Outreach worker; GTA). Another participant stated that “as far as policy and procedure go, [the decisions] really are up to the individual and the professional discretion of the staff member” (Outreach worker; Ontario).

Other participants, however, expressed apprehension to self-disclose. A participant, for instance, reported being “hesitant for people to be using their real names because then people are going to repeat their conversations to someone else,” and in a small community “people would know who that is right away” (Outreach worker; Ontario). In order to mitigate concerns over self-disclosure, some participants described using general agency logos as profile pictures and pseudonyms in order to provide online outreach to GB2M: “the photo is a red ribbon … so there is nothing specific about who I am” (Outreach worker; Ontario). Another participant explained:

This program was set up for multiple volunteers to use, what we’ve got, a common site, login ID; we’ve just really got agency logo, and a couple of agency promotional flyers, like, we’ve got PDFs of them up instead of pictures. (Outreach worker; GTA)

Generic photos were often utilized as a means to anonymize service providers’ identities: “I would create a fictitious profile of my body, but I would not give true elements of myself” (Outreach worker; GTA).

Participants also spoke about self-disclosure surrounding more personal information, such as HIV status or sexual practices. Participants recommended not disclosing personally identifiable information, such as HIV status, to service users. A participant indicated: “Nobody is ever expected to be out about their [HIV] status, for example, or anything like that” (Outreach worker; Ontario). However, the same participant also discussed the importance of determining self-disclosure (e.g., status) on a case-by-case basis:

I’m a gay man and I’m in a serodiscordant relationship. Sometimes I bring that up because it helps people see a perspective from maybe the negative guy’s point of view, fear of having sex with a poz guy. If I disclose that my partner is negative … sometimes that can help the conversation. (Outreach worker; Ontario)

Another participant indicated being aware of information placed on his profile:

When it comes to the big demographic stuff, age and sexual orientation and stuff, I have put that as being real; where I’ve had to be really, really careful, though, is to say what are you open to? I leave most of it open; I didn’t check off that I wasn’t into barebacking. So, somebody came back and said, oh, so you bareback. So I’ve got to be really careful about that sort of thing. (Outreach worker; Ontario)

Participants, overall, indicated various perspectives as to whether and what to disclose to service users. Agency policies and guidelines often left the decision to the individual outreach worker whereby opinions varied from choosing never to disclose, being transparent and disclosing some demographic information to making case-by-case assessments on whether it would benefit their practice.

The attitudes of participants echo previous research that has demonstrated that the decision of whether or not to self-disclose does not necessarily follow a universal gold-standard approach; rather, disclosure often emerges based on a personal understanding of how disclosure would benefit or harm the client and the therapeutic relationship [49]. Although this ethical issue has been identified and addressed in face-to-face practice, participants in the current study suggest that uncertainty also arises in online outreach. This may imply that comparable issues may emerge in both face-to-face and online interactions between service users and providers.

### Maintaining client confidentiality and anonymity

For service providers, assuring client confidentiality is an important component of helping service users feel safe and protected. However, in the digital world, there are limits in how confidentiality and anonymity can be maintained. For instance, there is often increased risk of information being shared across third parties and the inability that online platforms can secure complete confidentiality of clients. Therefore, an important component of online sexual health outreach for ASOs and CBOs is the capacity for service users to receive service provision through anonymous and confidential means: “the best part is the anonymity part [for service users]” (Outreach worker; Ontario). Participants identified confidentiality as a necessary skill-set of online outreach services: “discretion is a big thing. Knowing that somebody’s got the maturity to keep the information that is provided to them confidential” (Outreach worker; Ontario). Thus, the ability for online outreach workers to preserve matters as confidential and anonymous was an important ethical consideration for providing ethical and effective sexual health outreach.

Conducting online outreach may trigger concerns regarding confidentiality and anonymity: “there’s a lot of risk with online communication and interactions; you don’t know where it’s going to go, how it’s going to be shared” (Public health; GTA). Another participant reiterated this point, explaining that, “when you’re online, privacy and confidentiality, it’s a far more hazy [sic] concept than it is in person” (Outreach worker; Outside Ontario). Agencies demonstrated that service users’ confidentiality and anonymity was an important part of providing effective service provision: “We struggle as an agency just trying to figure out how to use text messages in a way that you can ensure that the person receiving the text message is in fact the person you intended to receive it.” (Manager; Ontario). Another participant articulated that “we still are kind of juggling and working to find out what those confidentiality limits look like and what can I do.” (Outreach worker; Outside Ontario).

In addressing ways to mitigate risks of confidentiality and anonymity, individual measures were taken among trained online outreach workers. For instance, a participant indicated how “all our volunteers and staff will sign confidentiality agreements” in order to protect clients’ anonymity (Outreach worker; GTA). Policies were also an important component in addressing confidentiality:

Those are some of the pieces that we try to incorporate in terms of the policies and procedures; dos and don’ts when you’re a volunteer or a co-ordinator because again you’re setting yourself up at risk, and you’re setting up the agency. (Outreach worker; GTA)

Referring specifically to the role of volunteers, a participant indicated that,

When they do join our leadership group, we have them sign this paper … it’s an agreement of what’s expected of you, and what you can expect from us … so conduct yourself in a way that is going to reflect positively. (Outreach worker; Outside Ontario)

Participants indicated various individual strategies employed by outreach workers and agencies to protect users’ confidentiality and anonymity when utilizing online sexual health outreach programs.

### Security and data storage measures of online information

Conducting online outreach introduces several ethical considerations regarding the security, safety and the storage of service user data. Beyond individual measures to address confidentiality concerns, as described in the theme above, breaches of confidentiality may occur through institutional programming, software and services that are run by administrators and nationally- or internationally-based organizations outside of the agency. Security, in this sense, is more than just individual means to protect confidentiality and anonymity (as explained in the theme above), but it is more so indicative of the way in which these larger software systems are able to store and protect client’s identifiable and sensitive information. Consequently, an inherent limitation to online sexual health outreach is that agencies cannot guarantee confidentiality, based on the terms of use and privacy settings of software and programs, in the same way as traditional in-person practice. A participant articulated that

There’s a lot of risk with online communication and interactions. You put that out there, it’s out there for good. You don’t know where it’s going to go, how it’s going to be shared. We know that email can bounce around to many servers and can be accessed in other ways. So there’s always a risk and it’s really just trying to find that sweet spot, the balance, in terms of when is the trade-off, in terms of privacy. (Outreach worker; Outside Ontario)

Participants addressed ways in which their agencies collect and store service user data to mitigate risk. Participants reported not keeping any identifiable client information: “We don’t save user names or anything like that, we take any identifying information off of the chat” (Outreach worker; Ontario). Another participant indicated that “We are just deleting the messages. We’re not keeping any record of them; we don’t keep user names, and we’re not printing chat transcripts…or reporting user names or IP addresses” (Outreach worker; Ontario). Participants reflected that sometimes the lack of documentation made online outreach difficult:

They [service users] truly are anonymous and we don’t have a lot of information. Sometimes it’s hard to communicate; it’s documented in an anonymous way because we don’t have the person’s identity, but we do need to capture what information we have provided. (Manager; Ontario)

Participants also reported being transparent with service users on how personal information is being stored and measures taken to protect their identity: “so just be aware that we’re not going to be sharing this and it’s locked up and it’s kept confidential but there is going to be a record of this conversation” (Outreach worker; Outside Ontario). Transparency with service users also “encourage[s] them [service users] that if there’s anything that they don’t want to disclose or they don’t want to talk about, it’s in their right to say ‘no, I don’t want to’ or find a way out of it” (Outreach worker; Outside Ontario).

Participants described the inevitability of risk, but tried to find ways to mitigate these concerns. A participant indicated that,

We’re constantly reviewing our approach and thinking about these [data storage and confidentiality] sorts of issues, in terms of the systems that we’re using, drafting the language, training and educating staff about how they use the technology to reduce risk as well because that’s really critical. (Public health; GTA)

Establishing secure online platforms and servers was also seen to help facilitate concerns over data collection and storage among service users. For instance, a participant discussed “trying to ensure that the platforms that we are using are private, that they’re going to protect confidentiality and that we can feel comfortable using those technologies every day” (Public health; GTA). Participants described the ways in which institutional level approaches, such as utilizing appropriate software and online data storage and information systems, applied precautionary measures to address limitations of online confidentiality and anonymity.

## Discussion

Our findings address ethical issues, raised by frontline staff and managers, of providing online sexual health outreach for GB2M through socio-sexual networking websites and mobile smartphone applications. We identified four themes in the data: (1) managing personal and professional boundaries with clients; (2) disclosing personal or identifiable information to clients; (3) maintaining client confidentiality and anonymity; and (4) security and data storage measures of online information. These four themes were identified by participants as important ethical issues that have emerged in their work with online sexual health outreach for GB2M.

Generally, participants reported similar ethical challenges to one another with respect to online sexual health outreach; but how they assessed and navigated these challenges were different based upon participants’ individual familiarity, comfort and experience with online outreach as well as organizational practice and policy. Some respondents, for instance, developed clear professional and personal boundaries, and preferred to remain completely anonymous by never using their real name or picture when conducting online outreach. Participants described using the organization’s logo, a pseudonym or other general identifiers to enhance anonymity. Others expressed transparency in their practice, seeing nothing problematic with, and even potential benefits to, providing service users with their name, picture, details about themselves (e.g. HIV status), or utilizing their “insider” status within the community. Yet, other participants remained uncertain on how to navigate boundaries or self-disclosure. For these participants, their decision was often made on a case-by-case basis, many of whom followed directives of their agencies that permitted individual discretion. The ethical complexity of online outreach elicits difficulties in establishing standardized policy guidelines and regulations to inform practice. Our findings illustrate that agencies are mindful of the ethical dimensions of online outreach, relying on the expertise of service providers to target service users’ needs, interests and experiences. The rapid expansion of online outreach may make it challenging for policies and guidelines to keep pace. Thus, an outreach workers’ personal experiences, social location and level of comfort and skills play an important role in informing ethical practice [50]. Outreach workers and managers acknowledge ethical ambiguity, and consider ways in which to monitor and inform ethical practice and risk management strategies.

Consequently, our findings show that ethics pertaining to issues of boundaries or self-disclosure may result in a more nuanced approach, a consequence of an outreach worker’s perspectives and assessment of their work and the clients whom they serve. A focus on individual decision-making and self-discretion to mitigate risk may be due to the lack of professional regulations among outreach workers. Individual outreach workers, as opposed to nurses who were also interviewed in the present study, are not clinically trained or members of a regulated professional body. Clinicians, such as social workers, nurses and psychologists, are bound by rules, standards and professional codes of ethics that provide much more comprehensive directives and standards on ways in which to provide ethically competent practice [51–53]. Consequently, being a member of an unregulated profession may not only influence individual frontline work, but may also impact agency guidelines, training, skill development and supervision provided to outreach workers. For example, the way in which participants identified and mitigated the ethical issues they saw as emerging in their practices may be entirely unique to those addressed in previous scholarship from the perspectives of social workers, psychologists and online psychotherapists. Formal online therapies have established concrete ways in which to monitor ethical issues that arise in practice between clients and practitioners. Most formal online technologies are managed through an encrypted, password-protected and secure server, utilized by trained healthcare professionals. Healthcare professionals are regulated by their professional body and have skills to identify and manage ethical issues associated with boundaries, disclosure, confidentiality, anonymity, and data security. In contrast, online sexual health outreach is a community-based approach and, thus, the personal discretion and skills of online outreach workers who are also community members is essential in providing ethical and effective services. Accordingly, online outreach workers may perceive ethical risks differently and seek different approaches to mitigate risk and enhance the value of their practice based on their comfort, familiarity and connections among GB2M.

The use of Internet-based platforms for online outreach has introduced confidentiality, anonymity and security concerns. With the proliferation of Internet-based technologies, there have been recurrent ethical inquiries regarding how personal information will be collected and stored without the risk of breaching confidentiality [9, 54, 55]. Formal e-therapy programs, for example, are conducted through firewall-encrypted servers that foster online safety. This is not the case for synchronous online sexual health outreach as described herein, which takes place within existing geo-locating and socio-sexual sites and applications to meet GB2M “where they are at,” an important part of community-based practice; measures of privacy and confidentiality cannot be guaranteed, as online tools are uniquely tied with technological devices, software or programs that have their own terms of use and security features [56, 57]. So, while online sexual health outreach programs may present similar challenges to in-person outreach in bathhouses and sex-on-site premises, conducting outreach online additionally relies on private/corporate infrastructure that may not necessarily be ideal for local service provision; rather, these software are expanding globally and issues pertaining to privacy and confidentiality may be susceptible to the decisions of larger companies, complying with the legal regulations or limitations of the country where the data is stored and macro-level interests.

Our findings illustrate that agencies employed various approaches to manage security and safety at the individual and structural levels: (1) training volunteers and outreach workers on how to effectively maintain confidentiality and anonymity; (2) the use of secure servers; (3) transparency with service users on limitations of confidentiality and anonymity; (4) data storage features/practices that strive to preserve client anonymity; and (5) developing confidentiality agreements for staff. In addition to software protection, agency measures were also taken to mitigate risks of privacy and safety, including deleting message histories, redacting identifiable information from records, not tracking IP addresses and deleting user names. Employing these security provisions indicates that outreach workers are mindful of the safety, confidentiality and anonymity of GB2M when conducting online outreach.

Although these ethical dimensions may not be easily resolved or completely eliminated, our findings demonstrate that outreach workers and managers are attuned to emerging ethical challenges. Participants considered the ethical implications of their work, and critically reflected on the ways in which to mitigate potential ethical ambiguity. Previous scholarly work has shown preliminary evidence of the benefits of online outreach for GB2M [42, 43]; accordingly, these ethical challenges should not prevent or hinder online practice but rather deepen our understanding of ethics to foster evidence-based strategies, such as training programs, appropriate supervision and risk management strategies to inform effective, safe and competent outreach. As a result, this does not necessarily entail limiting online outreach to specific geographical areas in order to protect anonymity. Rather, geographically remote areas are where online outreach may importantly benefit service users who have faced barriers in their access to face-to-face provision of sexual health services and information. Thus, outreach workers can begin to engage in meaningful dialogue at their agencies to consider ethical challenges of online sexual health outreach, and the diverse ways in which to manage ethical conflict if and when it does occur.

## Limitations

As a qualitative study, our aim was not to generalize, but to explore the perspectives of online outreach workers and managers with online sexual health outreach. Our findings likely present a more positive portrayal of experiences with these services, as we were only able to interview those who were interested, freely available, and largely still providing these services. Thus, findings of this study may not be representative of the experiences or perspectives of all individuals who practice online outreach. Our results might have been improved with more intentional comparison with existing in-person outreach programs and services, such as those provided in bathhouses and sex-on-site venues. One important missing narrative in this work is that of service users, who were not interviewed in the qualitative portion of this study [43]. Nevertheless, the study provides important and novel insights on ethical implications for outreach work among GB2M. There has been a paucity of empirically-based research that has explored online sexual health outreach among GB2M and, to our knowledge, none that has investigated the ethical considerations that may arise when using cyber communication for sexual health outreach.

## Implications

The purpose of this study is to recognize and address the potential ethical implications and risk management strategies that arise in online sexual health outreach. Our study may have implications for service providers, outreach workers, as well as community members, agency directors and managers, researchers, policy makers and bioethicists, in order to: (1) gain an understanding of the clinical and ethical implications of online HIV outreach for GB2M; (2) identify competencies and skills needed to conduct ethical online sexual health outreach; (3) foster dialogue and educational programming to discuss issues related to ethical conflict that may emerge in online sexual health practices; and (4) consider research and policy initiatives to establish evidence-informed guidelines and standards to facilitate clinical practice and agency structure with regard to online sexual health outreach among GB2M.

## Conclusions

This study may promote the development of ethical guidelines and frameworks to enhance online sexual health outreach among GB2M. Evidence-informed research is needed to explore the ethical challenges associated with online sexual health outreach, and to establish ways in which to competently and effectively mitigate potential ethical dilemmas.

## Abbreviations

GB2M: Gay, bisexual, two-spirit and other men who have sex with men
